# Kv1.3 Channel Blockade Improves Inflammatory Profile, Reduces Cardiac Electrical Remodeling, and Prevents Arrhythmia in Type 2 Diabetic Rats

**DOI:** 10.1007/s10557-021-07264-1

**Published:** 2021-10-08

**Authors:** Julián Zayas-Arrabal, Amaia Alquiza, Ainhoa Rodríguez-de-Yurre, Leyre Echeazarra, Víctor Fernández-López, Mónica Gallego, Oscar Casis

**Affiliations:** Department of Physiology, Faculty of Pharmacy, University of the Basque Country UPV/EHU, Paseo de la Universidad 7, 01006 Vitoria-Gasteiz, Spain

**Keywords:** Inflammation, Type 2 diabetes, Cardiac electrophysiology, Cytokines

## Abstract

**Purpose:**

Kv1.3 channel regulates the activity of lymphocytes, macrophages, or adipose tissue and its blockade reduces inflammatory cytokine secretion and improves insulin sensitivity in animals with metabolic syndrome and in genetically obese mice. Thus, Kv1.3 blockade could be a strategy for the treatment of type 2 diabetes. Elevated circulating levels of TNFα and IL-1b mediate the higher susceptibility to cardiac arrhythmia in type 2 diabetic rats. We hypothesized that Kv1.3 channel blockade with the psoralen PAP1 could have immunomodulatory properties that prevent QTc prolongation and reduce the risk of arrhythmia in type 2 diabetic rats.

**Methods:**

Type 2 diabetes was induced to Sprague-Dawley rats by high-fat diet and streptozotocin injection. Diabetic animals were untreated, treated with metformin, or treated with PAP1 for 4 weeks. Plasma glucose, insulin, cholesterol, triglycerides, and cytokine levels were measured using commercial kits. ECG were recorded weekly, and an arrhythmia-inducing protocol was performed at the end of the experimental period. Action potentials were recorded in isolated ventricular cardiomyocytes.

**Results:**

In diabetic animals, PAP1 normalized glycaemia, insulin resistance, adiposity, and lipid profile. In addition, PAP1 prevented the diabetes-induced repolarization defects through reducing the secretion of the inflammatory cytokines IL-10, IL-12p70, GM-CSF, IFNγ, and TNFα. Moreover, compared to diabetic untreated and metformin-treated animals, those treated with PAP1 had the lowest risk of developing the life-threatening arrhythmia *Torsade de Pointes* under cardiac challenge.

**Conclusion:**

Kv1.3 inhibition improves diabetes and diabetes-associated low-grade inflammation and cardiac electrical remodeling, resulting in more protection against cardiac arrhythmia compared to metformin.

**Supplementary Information:**

The online version contains supplementary material available at 10.1007/s10557-021-07264-1.

## Introduction

Among the diabetes-induced cardiac alterations is an electrical remodeling that modifies the expression and biophysical properties of different ion channels [1]. The main characteristic of diabetic cardiac electrical remodeling is the prolonged ventricular repolarization, reflected as a prolonged duration of the heart rate–corrected QT interval in the electrocardiogram (QTc). The prevalence of QTc prolongation varies from 30 to 50% in glucose-controlled type 2 diabetic (T2D) patients [2–4] and, more importantly, QTc prolongation is an independent predictor of all-cause mortality in treated T2D patients [5]. At the cellular level, by altering the expression of several ion channel proteins, diabetes lengthens the cardiac action potential duration [1], which increases the risk of developing ventricular arrhythmias and sudden death [6, 7].

Experimental, epidemiological, and clinical evidence causally links inflammation to the development of metabolic diseases like diabetes and/or the complications that emerge from these pathologies, particularly in the context of obesity and type 2 diabetes [8]. Inflammation plays a critical role in the development of type 2 diabetes. Diabetes associates with a whole-body low-grade inflammatory status characterized by elevated production of circulating cytokines such as IL-6, TNFα, IFNγ, TGFβ, MCP1, or IL-1b [9]. Among them, TNFα and IL-1b directly modulate the activity and expression of repolarizing cardiac ionic channels [10, 11]. Furthermore, elevated levels of TNFα and IL1b reduce the cardiac transient outward potassium current, prolong action potential duration, and are responsible for the increased susceptibility to arrhythmias in type 1 and 2 diabetic animals [10]. In a recent report, treatment with the IL-1b blocking agent anakinra suppressed ventricular arrhythmia refractory to conventional therapies [12].

The voltage-gated potassium channel Kv1.3 expresses in a number of tissues like neurons, lymphocytes, macrophages, or adipose tissue, participating in processes such as glucose homeostasis and cytokine production. Kv1.3^−/−^ mice have increased insulin sensitivity, and acute inhibition of Kv1.3 increases GLUT4 at the plasma membrane in *ob*/*ob* and *db*/*db* diabetic mice through a mechanism that involves downregulation of TNFα and IL-6 secretion [13]. In addition, Kv1.3 channel inhibition with toxins or synthetic peptidic analogs reduces cytokine secretion. Thus, incubation with margatoxin reduced TNFα and IL-6 secretion by adipose tissue in vitro [13] and ShK-189 treatment reduced TNFα production in adipose tissue in mice fed with obesity-induced diet [14]. Therefore, inhibition of Kv1.3 might be a pharmacological strategy for the treatment of metabolic, autoimmune, or chronic inflammatory diseases [14–16].

Several cytokines upregulated in type 2 diabetic patients [17] reduce the functional expression of cardiac repolarizing channels [10, 11] and are involved in the initiation and maintenance of cardiac arrhythmias in humans [12, 18]. Our group has recently reported that elevated circulating levels of TNFα and IL-1b are responsible for higher susceptibility to cardiac arrhythmia in type 2 diabetic rats [19]. We found that incubation with plasma extracted from diabetic animals prolonged action potential duration in control cardiomyocytes, causing a diabetes-like electrical phenotype in healthy cells, whereas adding IL-1b and TNFα receptor blockers to the diabetic plasma prevented the repolarization defects.

Thus, in the present work, we hypothesized that Kv1.3 channel inhibition can improve diabetic pathophysiology, including the cardiac electrical remodeling, by reducing the circulating levels of cytokines.

PAP1 (5-(4-Phenoxybutoxy) psoralen) is a small molecule that potently inhibits Kv1.3 with excellent selectivity over other Kv channels, transporters, receptors, and P450-dependent enzymes [20]. PAP1 has been used in preclinical studies in the context of inflammation and autoimmune processes [16, 21, 22]. In this work, we treated type 2 diabetic rats with PAP1 in order to determine whether Kv1.3 blockade improves the diabetes-induced low-grade inflammation, prevents the electrophysiological alterations, and thus protects against cardiac arrhythmia.

## Methods

### T2D Induction and Treatments

The investigation fulfilled the Spanish (RD 1201/2005) and European (D2003/65/CE and R2007/526/CE) rules for animal care used for experimental and other research purposes, and has been approved by the Ethics Committee for Animal Care of the Universidad del País Vasco (references CEBA/111a/2010 and CEBA/273M/2012). Eight-week-old male Sprague-Dawley rats (from the Animal Facility, UPV/EHU) were housed under a 12:12-h light:dark cycle in a temperature-controlled room (23°C).

Before starting the study, an IPIGTT (see below) was performed to ensure that the animals were not spontaneously insulin-resistant. Animals were randomized in four experimental groups: control; T2D; T2D treated with metformin (Sigma-Aldrich); and T2D treated with PAP1 (Oxchem). For the induction of diabetes, animals were fed on a high-fat diet (45% kcal from lipids) for 2weeks to induce insulin resistance, which was confirmed with an IPIGTT test. Then, a single intraperitoneal injection of 35 mg/kg streptozotocin (STZ) was applied to induce T2D (modified from ref. [23]) and the high-fat diet was maintained during the following 4 weeks. Thus, the full experimental period was 6 weeks in high-fat diet with a STZ injection at week 2. Forty-eight hours after STZ injection diabetes was confirmed by fasting blood glucose levels >126 mg/dl in two independent measurements. Treatments with PAP1 (intraperitoneal), metformin (drinking water), or vehicle started immediately after the diagnosis of diabetes and lasted 4 weeks.

In the clinical setting with diabetic patients, the dose of glucose-lowering drug often must be adjusted over time to achieve good glycemic control. This is also the case with our diabetic animals; therefore, the doses of PAP-1 and metformin were adjusted when needed. Previous bioavailability and effectiveness studies made in rats that received intraperitoneal injections of PAP1 showed that 6 and 10, but not 1 mg/kg body weight, yielded sustained plasmatic levels of PAP1 for up to 16–20 h post-injection [24]. Thus, we started from a daily ip. injection of 5 mg/kg and, when necessary, increased the dosage to up to 10 mg/kg for intensive glucose control. We compared PAP1 treatment with the first-choice drug for T2D metformin. In that group, animals initially received 50 mg/kg×day metformin in the drinking water. However, in the following weeks, most animals required 100 or 200 mg/kg×day to normalize glycemia.

Between 9 and 15 animals were randomized for each experimental group. Twelve animals were treated with PAP1 (T2D+PAP) and 15 diabetic animals were treated with metformin (T2D+Met). However, PAP1 or metformin monotherapy failed to control plasma glucose in two animals in each group and they were excluded from the study. The vehicles for PAP1 and metformin were Kremophor and drinking water respectively. Therefore, a group of 9 T2D animals received daily ip. injection of Kremophor, whereas another 9 diabetic animals had normal tap water. Since no difference between the two vehicle groups was observed, they were pooled and only one type 2 diabetic group is shown, T2D (*n*=18). Last, 15 control animals were used throughout the study in order to avoid possible biases due to time.

### Anesthesia

Anesthesia was induced by inhalation of 4% isoflurane and, when necessary, was maintained during the procedure with 2% isoflurane.

### Blood Measurements

After overnight fasting, animals were anesthetized and blood from the tail vein was collected. Blood glucose was determined with glucose reagent strips in a standard automated glucometer Accutrend Plus. Plasma insulin and cytokines were measured by ELISA kits and plasma cholesterol and triglycerides were determined using colorimetric test kits following manufacturer instructions.

### Combined Intraperitoneal Insulin and Glucose Tolerance Test

To avoid severe hypoglycemic episodes, we used a combined intraperitoneal insulin and glucose tolerance test (IPIGTT). Animals were fasted overnight and glucose (1g/kg) and insulin (1U/kg) were injected intraperitoneally. Blood was collected from the tail vein of anesthetized animals at 0, 15, 30, and 60 min after the injection and glucose was measured. For both the injections and the blood sample collection, animals were briefly anesthetized by inhalation of 4% isoflurane.

### In Vivo ECG Recordings

Two days before starting the experiments, permanent surgical stainless steel rings were placed, in the DII lead, under the skin of isoflurane-anesthetized animals. Subcutaneous electrodes can be easily connected through copper cables to a Biopac MP35 recording system controlled by the Biopac Pro software. Since the connection is minimally invasive, during the recordings, the animal was conscious in its cage, undisturbed and calm. Once a week, approximately 15 min of ECG was recorded. Corrected QT interval was calculated using the Fridericia formula: QTc=QT/(RR/1000)^0,33.

At the end of the 6-week experimental period, an arrhythmia susceptibility test consisting in 120 mg/kg of caffeine intraperitoneal and 50 μg/kg of dobutamine intravenous [25] was performed in isoflurane-anesthetized animals.

### Isolation of Ventricular Myocytes

Animals were anesthetized (50/7 mg/kg ketamine/xylazine, ip) and weighted. Then, the hearts were removed, weighted, mounted on a Langendorff apparatus, and perfused via the aorta at 37°C for 5 min with Tyrode solution of the following composition (mmol/l): NaCl 130, KCl 5.4, NaHCO_3_ 5.8, MgCl_2_ 1.05, CaCl_2_ 1.8, NaH_2_PO_4_ 0.42, dextrose 12, taurine 20, HEPES-Na 25, adjusted to pH 7.4 with NaOH, followed by a nominally Ca^2+^-free Tyrode solution for 10 min, and then by a Ca^2+^-free solution containing 1 mg/ml collagenase type II and 0.03 mg/ml protease Type XIV for 15 min. The enzymes were washed out with KB solution (in mmol/l): taurine 10, glutamic acid 70, creatine 5, succinic acid 5, dextrose 10, KH_2_PO_4_ 10, KCl 20, HEPES-K 10, EGTA-K 0.2, adjusted to pH 7.4 with KOH. Finally, the ventricles were excised and myocytes were isolated by mechanical agitation.

### Patch-Clamp Experiments

Action potentials were recorded at room temperature in enzymatically isolated ventricular myocytes, using the whole-cell configuration of the Patch-Clamp technique with an Axopatch200B amplifier. In the current-clamp configuration of the amplifier, 4-ms current pulses were applied at twice the threshold at a frequency of 2 Hz. Experimental protocols were controlled with the Clampex program and recordings analyzed with the Clampfit program of the pClamp 10.2 software.

Isolated myocytes were incubated for 24 h in serum-free DMEM and Media199 (4:1) containing 1% of penicillin-streptomycin. Media was supplemented with a cocktail of cytokines designed according to the results obtained in this work (Figure 3d, e; Supplementary table 1).

### Statistical Analysis

Data are presented as mean ± SEM. Each parameter in metformin-treated and PAP1-treated diabetic animals was compared at each time point with respect to control and to non-treated diabetic groups with one-way ANOVA. Since we compared more than two but less than six experimental groups, the Holm-Bonferroni post hoc test was applied. The statistical significance was set at *p*<0.05.

## Results

**Fig. 1:**
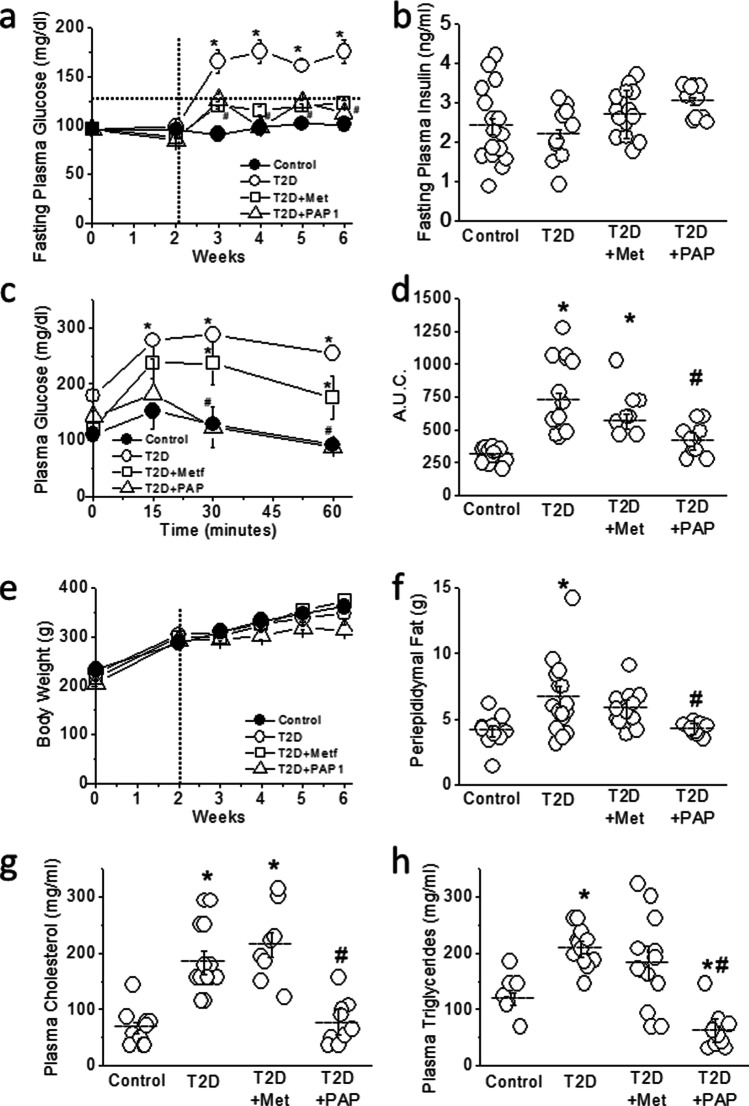
Metabolic parameters of type 2 diabetic (T2D) rats. **a** Weekly measurements of fasting plasma glucose. From week 0 to week 6, the diabetic animals were fed with a high-fat (45%) diet. The vertical dotted line indicates the moment of the STZ injection. The horizontal dotted line indicates the upper physiological value of blood glucose. Control animals were fed with standard chow and received an injection of vehicle. **b** Fasting plasma insulin levels at the end of the experimental period. **c** IPIGTT at the end of the experimental period. **d** Area under the curve (A.U.C.) from the data in **c**. **e** Evolution of body weight. **f** Abdominal fat at the end of the experimental period. **g**, **h** Plasma circulating levels of cholesterol and triglycerides. Horizontal bars represent Mean ± SEM. One-way ANOVA, followed by the Holm-Bonferroni post hoc test was applied. *p<0.05 with respect to control. ^#^*p*<0.05 with respect to T2D

Blood glucose levels were significantly lower in the metformin- and in the PAP1-treated versus non-treated T2D animals (*p*<0.01) and remained below the diabetic threshold throughout the experimental period. Thus, inhibition of Kv1.3 is an anti-hyperglycemic strategy as efficient as metformin treatment, since both PAP1 and metformin normalized fasting blood glucose during the diabetes period (Figure 1a).

At week 6, we measured fasting plasma insulin levels and performed an IPIGTT. Although plasmatic insulin levels were similar in the four experimental groups (Figure 1b), diabetic animals had insulin resistance (*p*<0.01; Figure 1c, d). PAP1 treatment, but not metformin, reduced insulin resistance in diabetic animals in a statistically significant manner (*p*<0.01 compared to T2D). Elevation of plasma glucose 15 min after the injection was significantly higher in non-treated and metformin-treated diabetic animals compared to control (*p*<0.01) and remained high at 60 min. Conversely, in the PAP1-treated animals, the increase in plasma glucose at 15 min was more modest and blood glucose levels were fully normalized at 30 min (Figure 1c, d). Moreover, metformin and PAP-1 therapy also normalized the HOMA-IR in T2D animals (Supplementary Figure 1).

At the end of the 6 weeks experimental period, control and diabetic animals (treated and untreated) had similar body weight (Figure 1e). However, T2D animals, fed on high-fat diet, showed a significant increase in abdominal fat compared to controls (*p*<0.01). Treatment with PAP1 but not with metformin prevented the accumulation of abdominal fat (Figure 1f). Similarly, only PAP1 treatment prevented the diabetes-induced dyslipidemia, characterized by high levels of cholesterol and triglycerides (Figure 1g, h).

Previous works showed that cardiac structural remodeling and contractile dysfunction appear after several months of the induction of diabetes [23]. So, as expected for a short-term diabetes, in our experimental model, we found no structural differences between control, diabetic, or PAP1-treated diabetic hearts (Supplementary Figure 2).

**Fig. 2:**
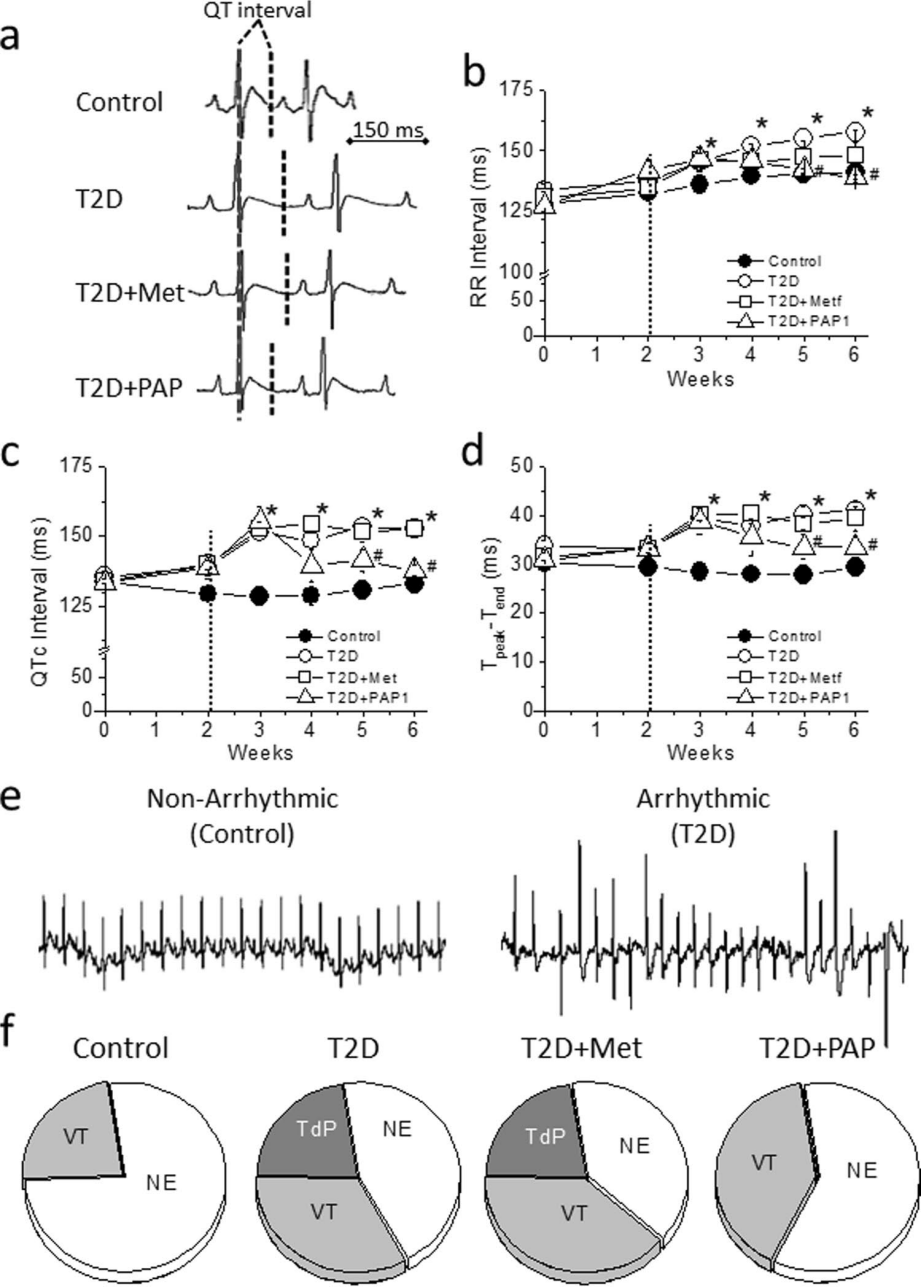
T2D-induced cardiac electrical remodeling and increased arrhythmia susceptibility. **a** Representative ECG recordings at the end of the experimental period. The dashed line shows the end of the T wave. **b**–**d** RR interval and main repolarization parameters, QTc, and T_peak-Tend_ throughout the 6-week experimental period. Only PAP1 treatment reversed the repolarization abnormalities. One-way ANOVA, followed by the Holm-Bonferroni post hoc test was applied. *p<0.05 with respect to control. ^#^*p*<0.05 with respect to T2D. **e** Traces of a non-arrhythmic and an arrhythmic ECG from a control and a T2D animal after caffeine/dobutamine challenge. **f** Incidence and severity of cardiac arrhythmias in control, T2D animals, and T2D animals treated with metformin or PAP1. No events (NE), ventricular tachycardia (VT), and Torsade de Pointes (TdP)

We next analyzed the effect of PAP1 in the diabetes-induced cardiac electrical remodeling. ECGs were recorded weekly in all experimental groups. As expected, compared to control, the ECG of T2D animals had lower heart rate reflected as higher RR interval duration (Figure 2a, b), as well as altered ventricular repolarization, shown as prolonged QTc interval and T_peak_-P_end_ duration (Figure 2a, c, d, p<0.01). Whereas both metformin and PAP1 normalized the heart rate in diabetic animals, only PAP1 restored QTc and T_peak_-T_end_ duration to control values. Thus, Kv1.3 inhibition prevented the diabetes-induced repolarization defects.

Prolonged QTc and T_peak_-T_end_ duration associates with higher risk of developing arrhythmia. As no spontaneous arrhythmic events were observed in the ECG recorded weekly, an arrhythmia-inducing protocol was performed. Figure 2e and f show that the incidence and severity of the arrhythmia were higher in T2D than in control animals after the caffeine/dobutamine challenge. Thus, 55% of the diabetic animals developed ventricular arrhythmia, in contrast to 23% of controls. Treatment with metformin did not reduce the susceptibility to arrhythmia, since metformin-treated rats had an incidence (61%) similar to that of untreated diabetic rats. However, treatment with PAP1 reduced the rate of arrhythmia (40%). Very interestingly, none of the animals treated with PAP1 developed the life-threatening ventricular tachycardia *Torsade de Pointes*, unlike the 22% of T2D or metformin-treated T2D. Thus, compared to metformin, PAP1 provided higher protection against the risk of developing arrhythmia.

**Fig 3:**
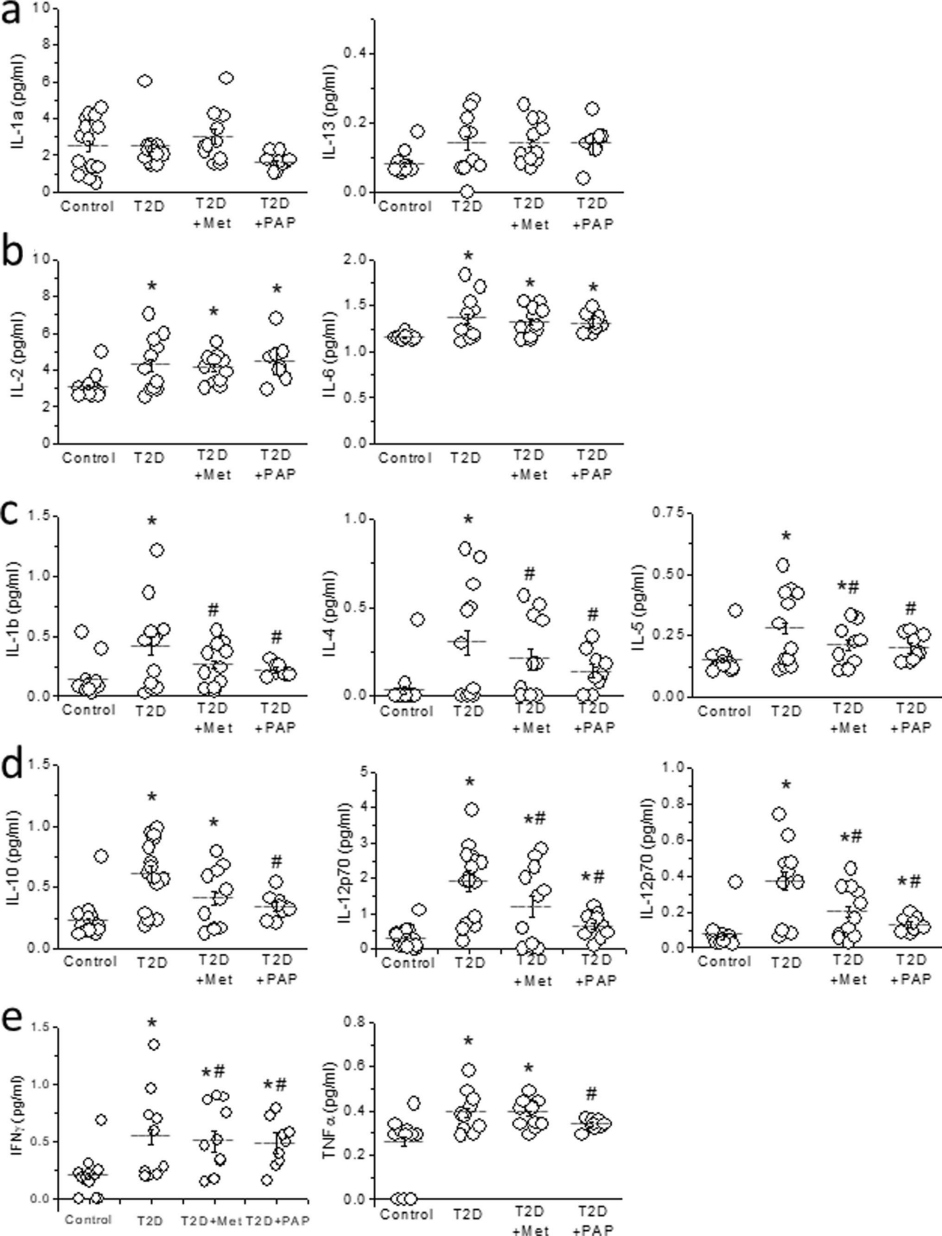
Plasmatic inflammatory profile in T2D. **a** Plasma levels of IL-1a and IL-13 were not modified in any experimental group. **b** T2D increased IL-2 and IL-6 levels, which were not modified by any treatment. **c** T2D increased plasma levels of IL-1b, IL-4, and IL-5, and were normalized by treatment with metformin or PAP1. **d** T2D increased plasmatic levels of IL-10, IL-12p70, and GM-CSF, which were slightly reduced by metformin and corrected by PAP1. **e** IFNγ and TNFα were higher in T2D animals, not modified by metformin and corrected by PAP1 treatment. Horizontal bars represent mean ± SEM. One-way ANOVA, followed by the Holm-Bonferroni post hoc test was applied. *p<0.05 with respect to control. #*p*<0.05 with respect to T2D

Type 2 diabetes is associated with a low-grade inflammation and we wondered whether Kv1.3 inhibition could improve it. Thus, at the end of the experimental period, we measured plasma levels of twelve cytokines. Except for the case of IL-1a and IL-13 (Figure 3a), diabetic animals had significantly elevated levels of the tested cytokines: IL-2, IL-6, IL-1b, IL-4, IL-5, IL-10, IL-12p70, GM-CSF, IFNγ, and TNFα compared to control (Figure 3b–e, p<0.01). Treatment with metformin or PAP1 had no effect on IL-2 and IL-6 (Figure 3b), whereas both drugs had similar effect reducing IL-1b, IL-4, and IL-5 (Figure 3c) compared to those of untreated diabetic animals (*p*<0.01). However, regarding the other cytokines, metformin and PAP1 behaved differently. Plasma levels of IL-10, IL-12p70, and CM-CSF were lower in metformin-treated rats than in T2D animals, but still elevated compared to controls. On the contrary, PAP1 treatment restored these cytokines to control values (Figure 3d). Moreover, metformin did not improve, whereas PAP1 normalized IFNγ and TNFα secretion (Figure 3e).

Our electrocardiographic results showed that PAP1 treatment normalized QTc and T_peak_-T_end_ prolongation, thus reducing the risk of developing TdP under cardiac challenge. Therefore, we wanted to determine if this normalization of the repolarization could be caused by the improvement in the inflammatory profile. To test this hypothesis, we recorded action potentials in ventricular myocytes isolated from control animals. Cells were incubated 24h with specific cocktail of cytokines, designed according to the results shown in Figure 3d and e, in order to mimic the inflammatory profile of control, T2D, metformin-treated, and PAP1-treated animals. We focused on those cytokines modified differentially by metformin and PAP1, which are IL-10, IL-12p70, GM-CSF, IFNγ, and TNFα, and prepared the cocktails based on the plasma concentrations obtained in each experimental group (Supplementary table 1).

**Fig. 4:**
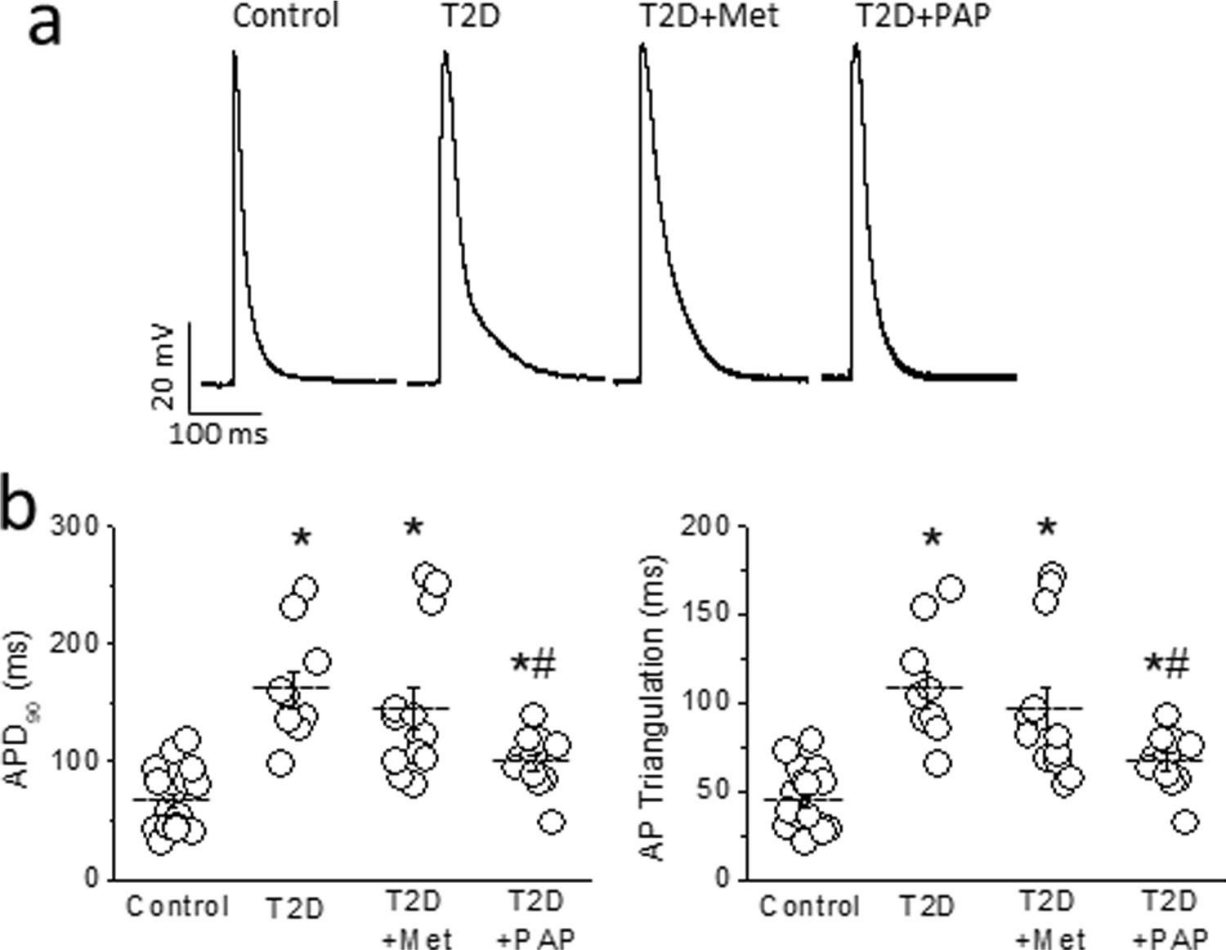
Different circulating concentrations of cytokines differentially modulate cardiac repolarization. **a** Action potentials recorded in ventricular myocytes isolated from healthy animals after 24-h incubation with one of four cocktails of cytokines at the concentrations measured in plasma of Control, T2D, T2D+Met, and T2D+PAP animals. **b** Incubation with the cytokine cocktail containing IL-10, IL-12p70, GM-CSF, IFNγ, and TNFα, at the concentrations measured in plasma of T2D animals prolonged action potential duration at 90% of repolarization (APD_90_) and increased action potential triangulation. PAP1 treatment but not metformin partially prevented these repolarization abnormalities. Horizontal bars represent mean ± SEM. *n*=18, 10, 13, and 11 cells from 7, 7, 4, and 8 animals in control; T2D, T2D+Met, and T2D+PAP respectively. One-way ANOVA, followed by the Holm-Bonferroni post hoc test was applied. *p<0.05 with respect to control. ^#^*p*<0.05 with respect to T2D

Action potentials (APs) recorded in cells incubated with the control cocktail were normal and showed the expected duration at the 90% of repolarization (APD_90_) and triangulation (Figure 4). Incubation of control cells with the T2D cocktail significantly (*p*<0.01) prolonged (APD_90_) and AP triangulation, and similar results were obtained with the T2D+Met cocktail. However, in cells incubated with the T2D+PAP cocktail, although APD_90_ and AP triangulation were still longer compared to those of the control cocktail group (*p*<0.01), they were significantly shorter compared to those of the T2D or T2D+Metf groups (*p*<0.01). In summary, different concentrations of cytokines differentially modulate the cardiac action potential shape and duration.

## Discussion

In this work, we examined the blockade of Kv1.3 as a potential target in the treatment of type 2 diabetes. Treatment with PAP1, but not metformin, improves insulin sensitivity and reverses cardiac electrical remodeling. Since diabetic animals had an altered cytokine profile that is more effectively normalized by PAP1 than by metformin, our results suggest that PAP1 normalized the metabolic parameters and the electrical alterations by reducing the secretion of several inflammatory cytokines.

In type 2 diabetic rats, treatment with PAP1 normalized blood glucose levels, insulin resistance, abdominal fat accumulation, and dyslipidemia. These results are in line with those observed in Kv1.3^−/−^ mice, as well as in those obtained in obese mice and mice fed with obesity-inducing diet after treatment with other Kv1.3 inhibitors, like ShK-186 and Margatoxin [14, 26].

Adipose tissue expansion has been proposed as the origin of the low-grade inflammatory state in type 2 diabetes, where blood levels of more than 20 cytokines are high [9, 27, 28]. This low-grade inflammation plays a critical role in the development and pathophysiology of T2D [8]. Our metabolic model of type 2 diabetes did not affect total body weight. However, after 6 weeks of feeding on high-fat diet, animals had significantly more abdominal fat than controls. Also at week 6, we found elevated secretion of ten cytokines, IL-2, IL-6, IL-1b, IL-4, IL-5, IL-10, IL-12p70, GM-CSF, IFNγ, and TNFα. Our T2D model has normal insulin and elevated cytokine levels, consistent with the results reported in obese mice, where adiposity triggers an inflammatory response prior to the increase of fasting insulin levels [29]. Our experiments cannot distinguish if the reduced abdominal fat tissue is the result of the altered cytokine profile or rather a direct effect of PAP-1 on adipose tissue. However, in mice fed on obesity-inducing diet, the Kv1.3 blocker ShK-186 reduced body weight and adiposity by increasing energy expenditure upon activation of the brown adipose tissue, but not the white, and by reducing the inflammation of abdominal fat [14]. Thus, in our diabetic animals, PAP1 treatment prevented the inflammation due, at least in part, to the reduction in the adipose tissue.

The Kv1.3 channel modulates the resting membrane potential and therefore, the activation, differentiation, and cytokine production in response to injury in macrophages, and B and T lymphocytes [30–34]. We have also observed Kv1.3 channel expression in the heart (Supplementary Figure 3). Since Kv1.3 blockade reduces the cytokine secretion by these cells [14, 33, 35], in this work, we used the selective Kv1.3 blocker PAP1 as immunomodulator. As expected, treatment of T2D rats with PAP1 significantly reduced the plasma levels of eight cytokines: IL-1b, IL-4, IL-5, IL-10, IL-12p70, GM-CSF, IFNγ, and TNFα. These are circulating cytokines; therefore, it is not possible to discriminate if they originate from adipose tissue and/or inflammatory cells like lymphocytes or macrophages.

Prolonged QTc interval is present in up to 66% of type 1 and 51% of type 2 diabetic patients with good glycemic control, and is recognized as an independent predictor of all-cause mortality in T2D patients [2, 5]. As expected for an animal model of diabetes, our T2D rats had prolonged repolarization and treatment with PAP1 efficiently prevented this lengthening. Moreover, as a result, PAP1-treated diabetic animals had less susceptibility to develop arrhythmia, particularly TdP, under cardiac challenge. Thus, Kv1.3 blockade with PAP1 prevented the diabetes-induced cardiac electrical remodeling.

It has been reported that metformin reduced the serum levels of TNFα in rodents fed with high-fat diet [36, 37]. In our T2D animals, metformin did not prevent fat accumulation and was not as effective as PAP1 in reducing the secretion of IL-10, IL-12p70, GM-CSF, IFNγ, and TNFα. This could explain why metformin treatment did not prevent the electrocardiographic alterations and did not reduce the susceptibility to arrhythmia in the caffeine/dobutamine challenge.

Cardiac dysfunction induces splenic cells to mobilize and accumulate in the heart [38]. It was described [10] and has been recently confirmed [39] a higher content of inflammatory cells in the diabetic myocardium compared to control. However, a recent study [19] points to a role for systemic over local inflammation on the cardiac electrical remodeling. The experiments with the cocktails of cytokines presented here reinforce the relevance of systemic inflammation. Control cardiomyocytes incubated with a cytokine cocktail that mimicked the plasma of diabetic animals had prolonged action potential duration, thus adopting a diabetes-like electrical phenotype. Incubation with the cytokine cocktail that mimicked the plasma of metformin-treated rats failed to prevent the increase in APD. Conversely, the cocktail that replicated the plasma of PAP1-treated diabetic animals partially prevented the prolonged APD. These ex vivo experiments show that modulation of cytokine levels directly modulates cardiac repolarization.

### Study Limitations

The main limitation of this study is that in diabetic patients, the cytokine profile may be more complex than the one we have reported in diabetic rats. In the human blood, there are a number of circulating cytokines. Most of them are upregulated in situations of inflammation, but not all are reduced after treatment. In humans, treatment with Kv1.3 inhibitors might alter in a different way the levels of important cytokines.

Elevated IL1b is responsible for the proarrhythmic cardiac remodeling in type 1 diabetic animals [10] and is elevated in our model of T2D. However, both metformin and PAP1 normalize IL-1b levels, whereas only PAP1 prevents the remodeling. This suggests that particular combinations of cytokines induce distinct functional changes in tissues.

Finally, Kv1.3 channel inhibition reduces the activity of in macrophages, B and T lymphocytes, and neutrophils due to depressed polarization and consequent decreased Ca^2+^ signaling [15, 30–35, 40]. Thus, a downside effect of inhibition of these channels could be an attenuation of the immune response, which could be dangerous in acute conditions of trauma.

## Conclusion

Kv1.3 inhibition is an interesting potential therapeutic target for the treatment of type 2 diabetes. It not only normalizes glycemia and insulin resistance, but also improves low-grade inflammation and cardiac electrical remodeling, which results in more protection against cardiac arrhythmia than with the first choice drug metformin. This could be of special interest in diabetic patients with associated complications, such as heart failure, that can amplify the risk of arrhythmia.

## Supplementary Information

Below is the link to the electronic supplementary material.

Supplementary file1 (DOCX 14 KB)

Supplementary file2 (DOCX 14 KB)

Supplementary Figure 1 (PNG 158 KB)

High Resolution Image (TIF 47 KB)

Supplementary Figure 2 (PNG 1421 KB)

High Resolution Image (TIF 317 KB)

Supplementary Figure 3 (PNG 205 KB)

High Resolution Image (TIF 77 KB)

## Data Availability

The datasets generated during during the current study will be shared on reasonable request to the corresponding author.
