# Supplementary material for: Kv1.3 Channel Blockade Improves Inflammatory Profile, Reduces Cardiac Electrical Remodeling, and Prevents Arrhythmia in Type 2 Diabetic Rats
Source: Cardiovasc Drugs Ther. 2021 Oct 8;37(1):63–73. doi: 10.1007/s10557-021-07264-1 (PMC9834174; doi:10.1007/s10557-021-07264-1)
Supplement: Supplementary file 1 — Supplementary file1 (DOCX 14 KB) [file 10557_2021_7264_MOESM1_ESM.docx]

**Kv1.3 channel blockade improves inflammatory profile, reduces cardiac electrical remodeling and prevents arrhythmia in type 2 diabetic rats.**

*Cardiovasc Drug Ther*

Julián Zayas-Arrabal, Amaia Alquiza, Ainhoa Rodríguez-de-Yurre, Leyre Echeazarra, Víctor Fernández-López, Mónica Gallego, Oscar Casis.

**Address for correspondence:** Dr. Oscar Casis. Departamento de Fisiología, Facultad de Farmacia, Universidad del País Vasco, Paseo de la Universidad 7, 01006 Vitoria-Gasteiz, Spain. Tf: +34 945013033. Fax: +34 945013327. Email: [oscar.casis@ehu.eus](mailto:oscar.casis@ehu.eus)

**Supplementary Table 1. Composition of the cytokines cocktails for myocytes incubation.** Four cocktails were prepared containing the cytokines modified differentially by metformin and PAP1 at the concentrations measured in plasma of the four experimental groups: Control, T2D, T2D+Met and T2D+PAP.

|  | **IL10 [ng/ml]** | **IL12 [ng/ml]** | **GM-CSF [ng/ml]** | **IFNγ [ng/ml]** | **TNFα [ng/ml]** |
| --- | --- | --- | --- | --- | --- |
| **Control** | 230 | 85 | 45 | 235 | 275 |
| **T2D** | 600 | 1730 | 340 | 585 | 400 |
| **T2D+Met** | 400 | 1060 | 170 | 545 | 380 |
| **T2D+PAP** | 340 | 460 | 100 | 395 | 270 |
