# Supplementary material for: Kv1.3 Channel Blockade Improves Inflammatory Profile, Reduces Cardiac Electrical Remodeling, and Prevents Arrhythmia in Type 2 Diabetic Rats
Source: Cardiovasc Drugs Ther. 2021 Oct 8;37(1):63–73. doi: 10.1007/s10557-021-07264-1 (PMC9834174; doi:10.1007/s10557-021-07264-1)
Supplement: Supplementary file 2 — Supplementary file2 (DOCX 14 KB) [file 10557_2021_7264_MOESM2_ESM.docx]

**Kv1.3 channel blockade improves inflammatory profile, reduces cardiac electrical remodeling and prevents arrhythmia in type 2 diabetic rats.**

*Cardiovasc Drug Ther*

Julián Zayas-Arrabal, Amaia Alquiza, Ainhoa Rodríguez-de-Yurre, Leyre Echeazarra, Víctor Fernández-López, Mónica Gallego, Oscar Casis.

**Address for correspondence:** Dr. Oscar Casis. Departamento de Fisiología, Facultad de Farmacia, Universidad del País Vasco, Paseo de la Universidad 7, 01006 Vitoria-Gasteiz, Spain. Tf: +34 945013033. Fax: +34 945013327. Email: [oscar.casis@ehu.eus](mailto:oscar.casis@ehu.eus)

**Supplementary Figure 1. PAP1 and metformin normalized HOMAi in diabetic rats.** HOMA index of Control, T2D, T2D+Met and T2D+PAP animals at the end of the experimental period (week 6). In diabetic animals, treatment with PAP1 or with metformin normalized the HOMA index. HOMAi = [fasting glucose (mg/dL) x Fasting insulin (mUI/L)] / 22.5. Horizontal bars represent Mean ± SEM. One-way ANOVA, followed by the Holm-Bonferroni post-hoc test was applied. *p<0.05 with respect to control.

**Supplementary Figure 2. Type 2 diabetic animals do not show cardiac hypertrophy or fibrosis. a)** Heart weight to tibia length ratio were similar in Control, T2D and T2D+PAP animals. Hearts were briefly plunged in PBS, drained by gentle squeeze and weighted. Right tibias were dissected and the bone was measured with caliper rule. Horizontal bars represent Mean ± SEM. **b)** Representative Masson’s trichrome stainings in ventricles from Control, T2D and T2D+PAP animals. Hearts do not show interstitial (left) or perivascular (right) fibrosis.

**Supplementary Figure 3. Kv1.3 expresses in cardiac tissue.** Kv1.3 protein levels in heart and liver from control animals. Samples were lysed in RIPA buffer and 120 μg of total protein were fractionated on 8% SDS-polyacrylamide gels and transferred to nitrocellulose membranes. Blots were incubated with primary antibodies anti-Kv1.3 (1:500, Sigma-Aldrich) and anti-GAPDH (1:1000; Sigma-Aldrich). Secondary antibodies were conjugated with Horseradish Peroxidase and blots were developed using enhanced chemiluminescence (West Pico, Thermo Scientific). Kv1.3 channel is expressed in cardiac tissue but not in liver.
