# Supplementary figures and images for: Kv1.3 Channel Blockade Improves Inflammatory Profile, Reduces Cardiac Electrical Remodeling, and Prevents Arrhythmia in Type 2 Diabetic Rats
Source: Cardiovasc Drugs Ther. 2021 Oct 8;37(1):63–73. doi: 10.1007/s10557-021-07264-1 (PMC9834174; doi:10.1007/s10557-021-07264-1)

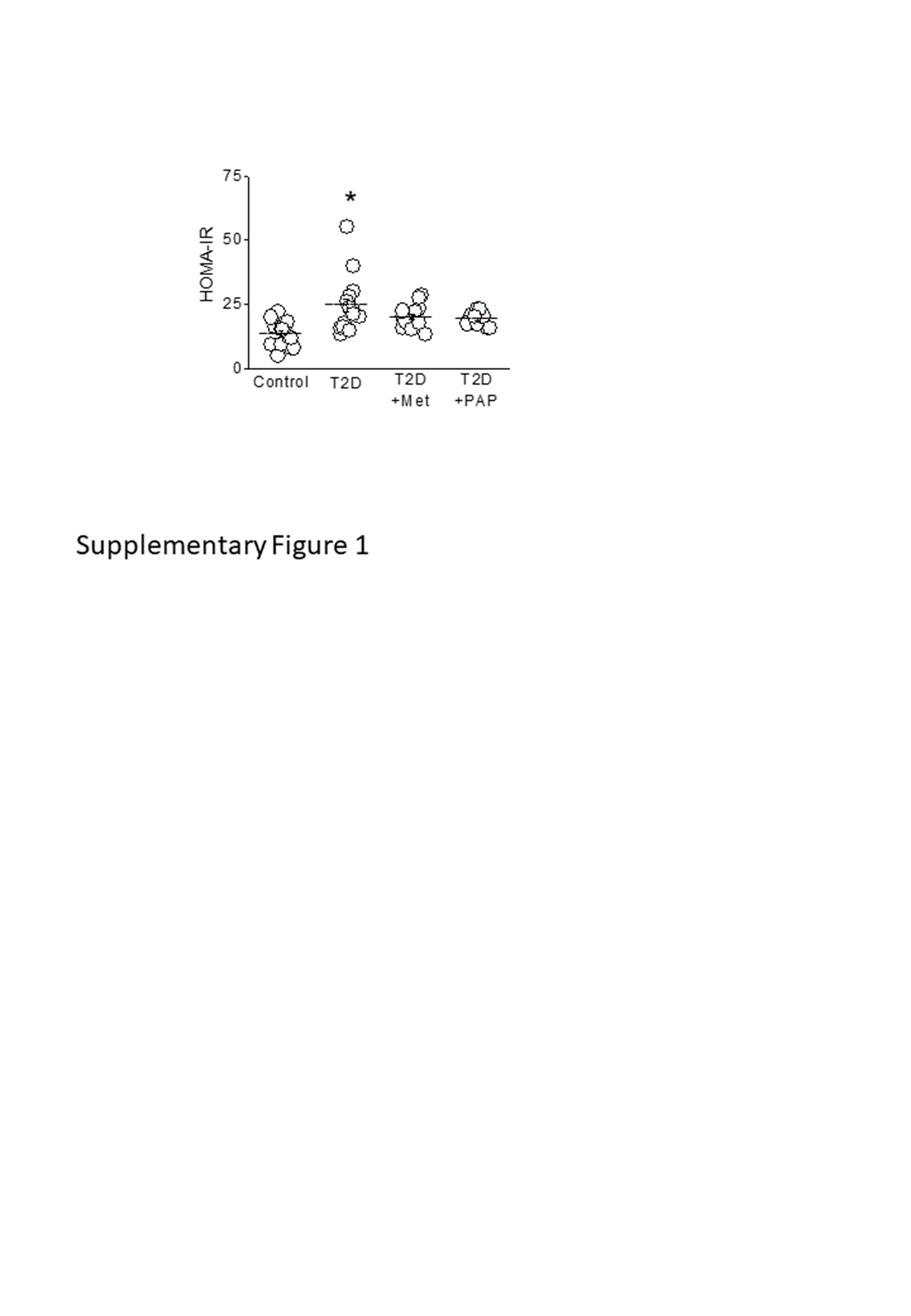

Supplement: Supplementary file 3 — (PNG 158 KB) [file 10557_2021_7264_Fig5_ESM.png]

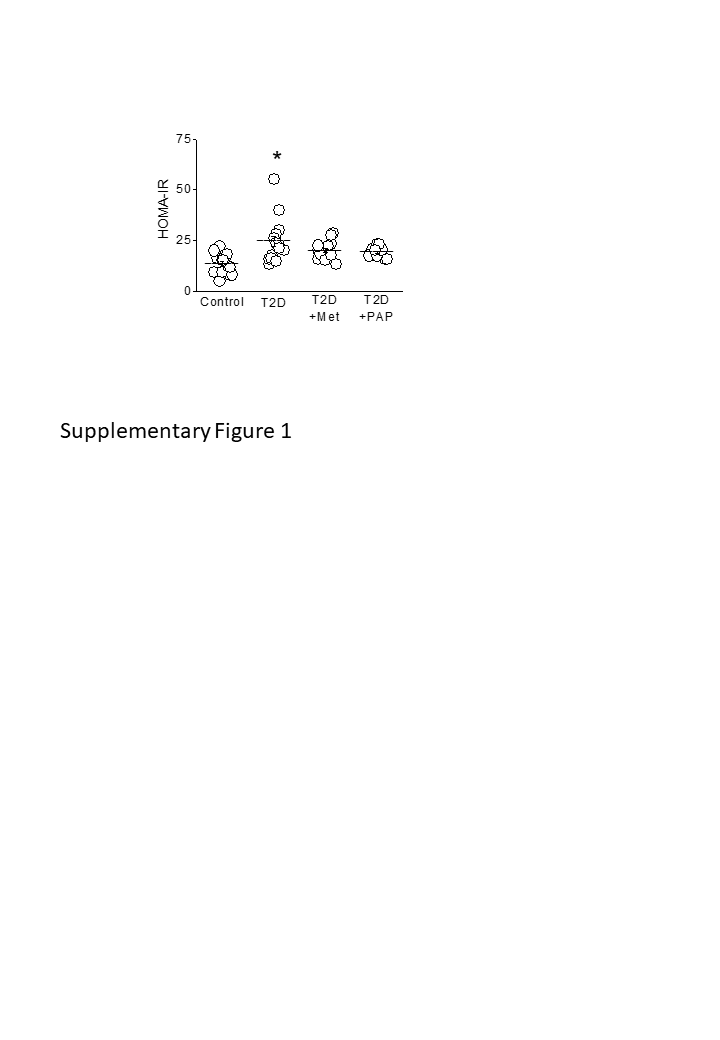

Supplement: Supplementary file 4 — High Resolution Image (TIF 47 KB) [file 10557_2021_7264_MOESM3_ESM.tif]

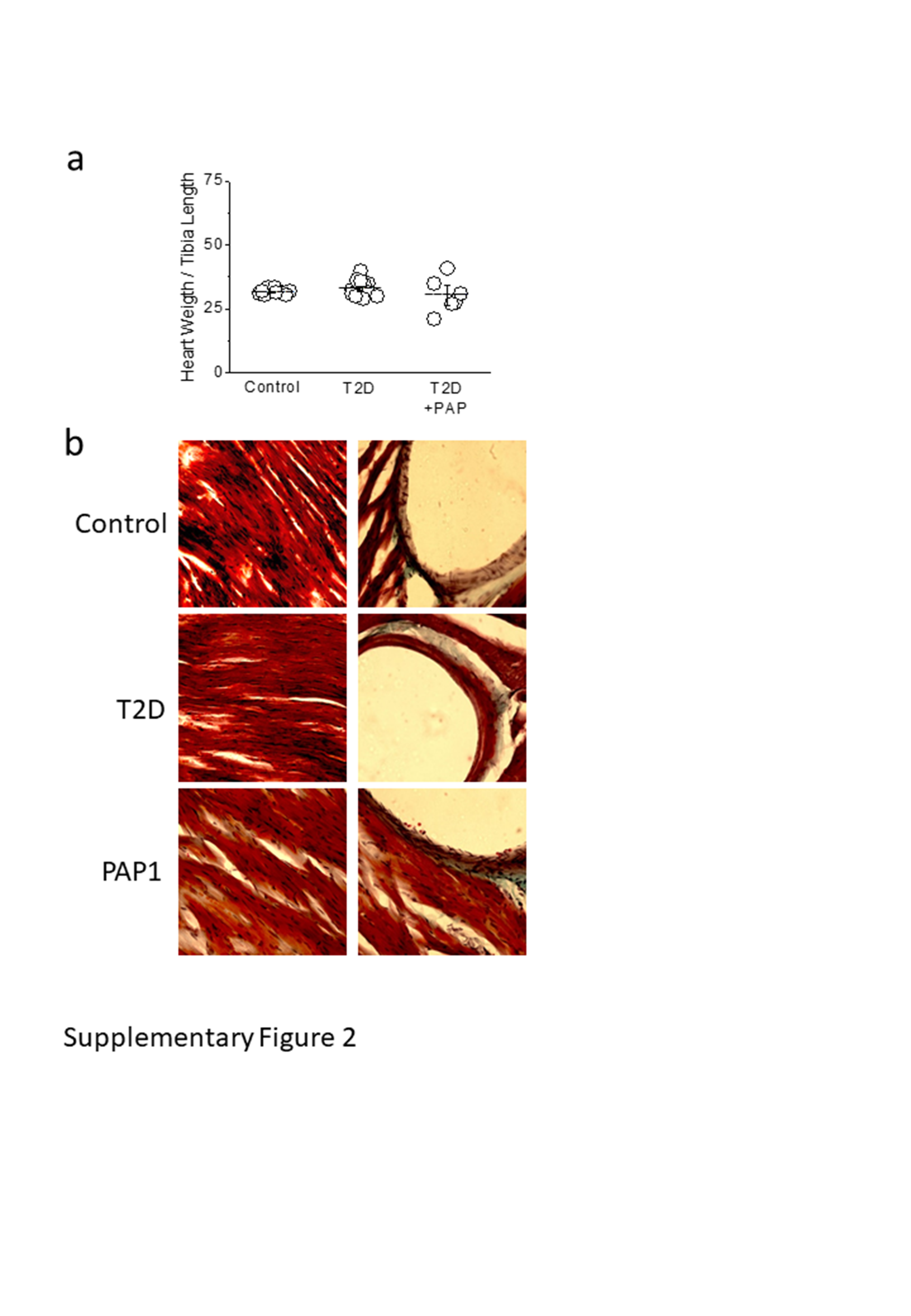

Supplement: Supplementary file 5 — (PNG 1421 KB) [file 10557_2021_7264_Fig6_ESM.png]

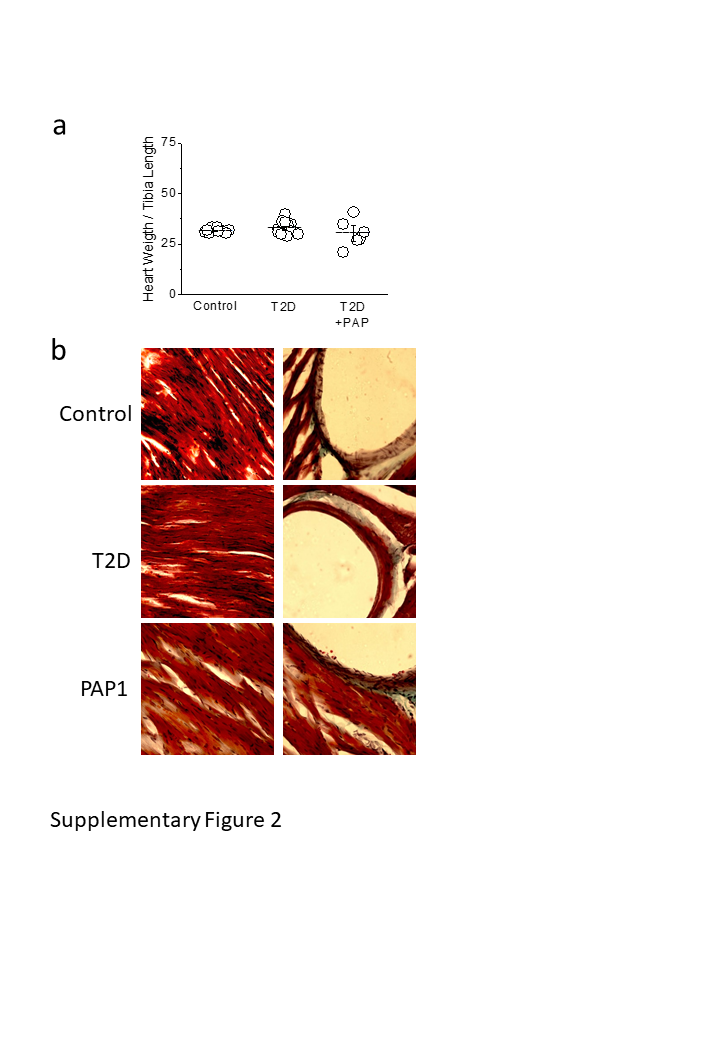

Supplement: Supplementary file 6 — High Resolution Image (TIF 317 KB) [file 10557_2021_7264_MOESM4_ESM.tif]

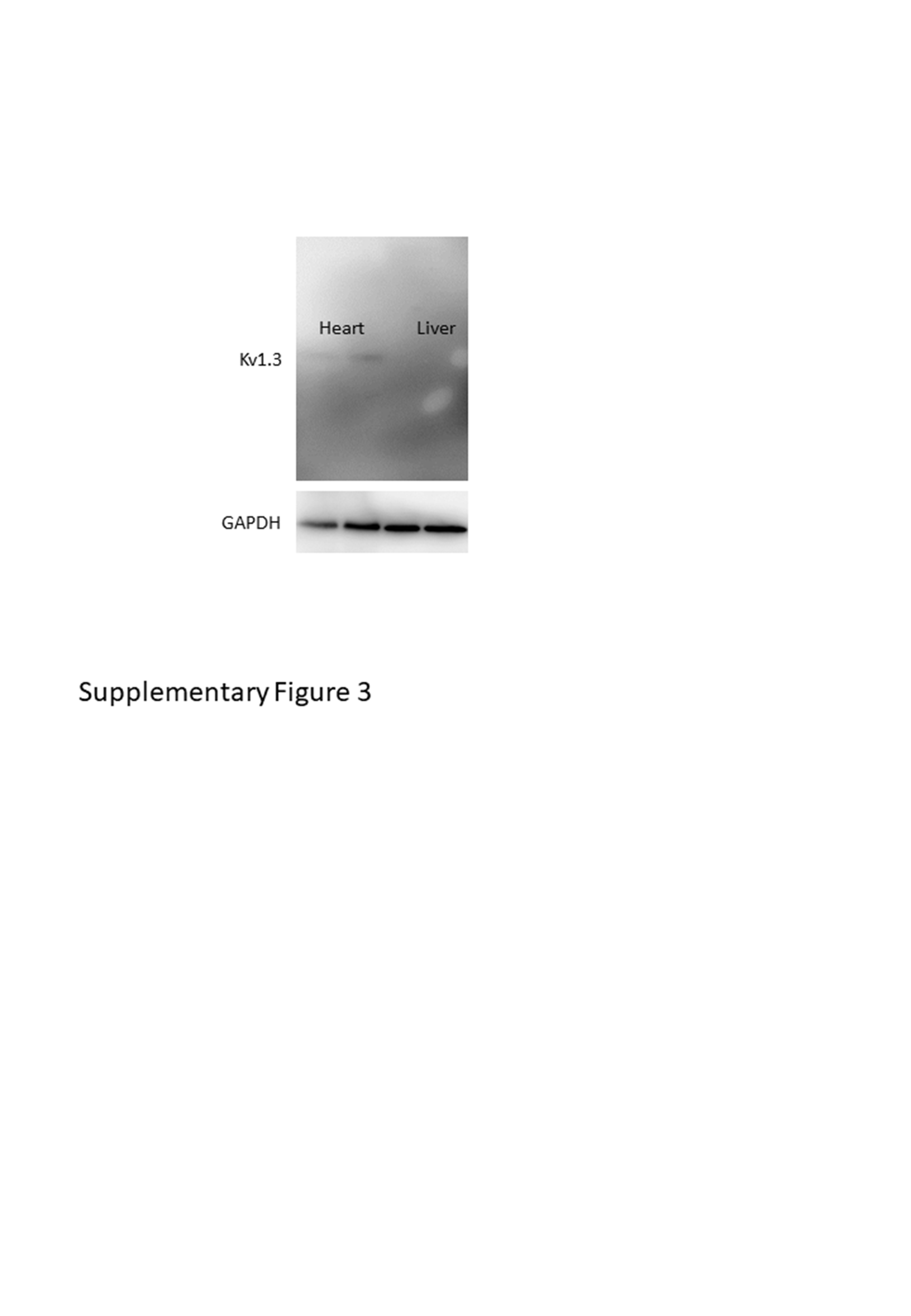

Supplement: Supplementary file 7 — (PNG 205 KB) [file 10557_2021_7264_Fig7_ESM.png]

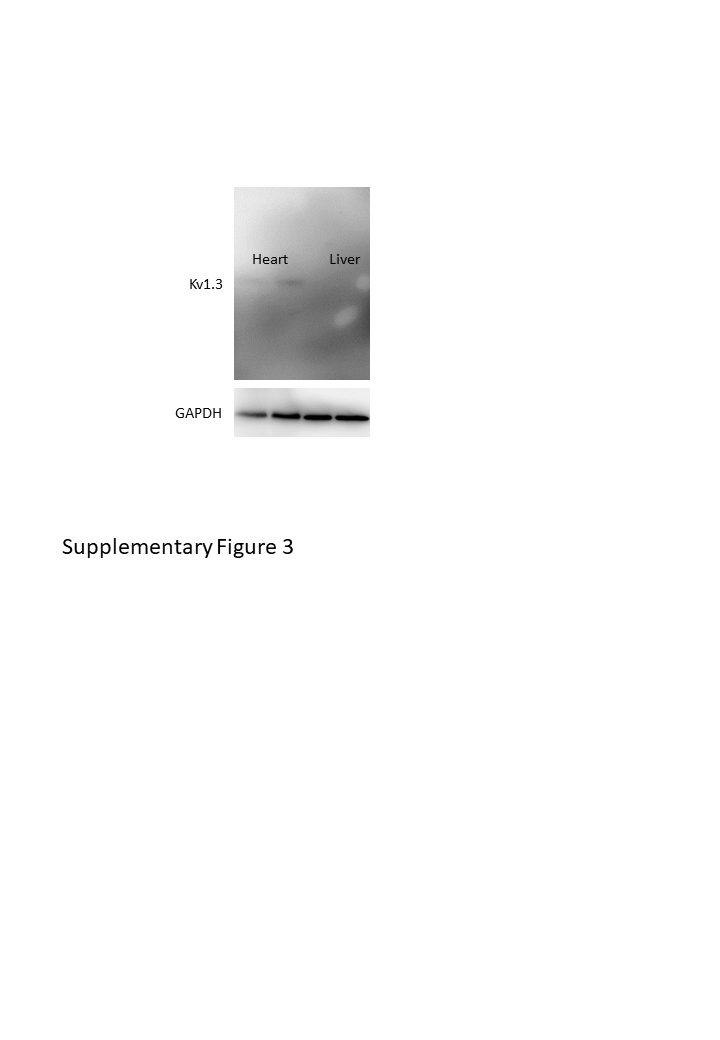

Supplement: Supplementary file 8 — High Resolution Image (TIF 77 KB) [file 10557_2021_7264_MOESM5_ESM.tif]
